# Nonverbal sound processing in semantic dementia: A functional MRI study

**DOI:** 10.1016/j.neuroimage.2012.02.045

**Published:** 2012-05-15

**Authors:** Johanna C. Goll, Gerard R. Ridgway, Sebastian J. Crutch, Frederic E. Theunissen, Jason D. Warren

**Affiliations:** a Dementia Research Centre, UCL Institute of Neurology, University College London, London, United Kingdom; b Wellcome Trust Centre for Neuroimaging, UCL Institute of Neurology, University College London, London, United Kingdom; c Psychology Department, University of California, Berkeley, USA

**Keywords:** Dementia, Semantic dementia, fMRI, Auditory perception, Auditory object

## Abstract

Semantic dementia (SD) is a unique neurodegenerative syndrome accompanied by relatively selective loss of the meaning of objects and concepts. The brain mechanisms that underpin the syndrome have not been defined: a better understanding of these mechanisms would inform our understanding of both the organisation of the human semantic system and its vulnerability to neurodegenerative disease. In this fMRI study, we investigated brain correlates of sensory object processing in nine patients with SD compared with healthy control subjects, using the paradigm of nonverbal sound. Compared with healthy controls, patients with SD showed differential activation of cortical areas surrounding the superior temporal sulcus, both for perceptual processing of spectrotemporally complex but meaningless sounds and for semantic processing of environmental sound category (animal sounds versus tool sounds). Our findings suggest that defective processing of sound objects in SD spans pre-semantic perceptual processing and semantic category formation. This disease model illustrates that antero-lateral temporal cortical mechanisms are critical for representing and differentiating sound categories. The breakdown of these mechanisms constitutes a network-level functional signature of this neurodegenerative disease.

## Introduction

Semantic dementia (SD) is a highly characteristic syndrome of relatively selective temporal lobe degeneration with progressive loss of the meaning of objects and concepts. It is the paradigmatic disorder of human semantic memory: the memory system by which we acquire, represent and store knowledge about the world. SD is of high neurobiological importance, firstly because it presents a striking model of selective involvement of functional brain systems by neurodegenerative pathology, and secondly, because it potentially informs our understanding of the brain organisation of semantic processing. Semantic deficits in SD have been most thoroughly characterised in the language domain but are also well documented in the visual, auditory and other sensory modalities (Warrington, 1975; Bozeat et al., 2000; Coccia et al., 2004; Goll et al., 2010a; Luzzi et al., 2007; Piwnica-Worms et al., 2010). SD preferentially targets a brain network centred on the anterior temporal lobes (Chan et al., 2001; Rohrer et al., 2010; Seeley et al., 2009), suggesting that this network plays a crucial role in semantic processing and in the pathogenesis of the SD syndrome. However, whilst SD has been studied extensively from a neuropsychological perspective (Bonner et al., 2010; Hodges and Patterson, 1996; Hodges and Patterson, 2007), the underlying pathophysiological mechanisms of SD remain poorly understood (Fletcher and Warren, 2011).

Our knowledge about the world is initially derived via particular sensory channels; it would be surprising a priori if brain mechanisms of semantic processing did not in some way reflect sensory characteristics. Both neuropsychological studies (Warrington, 1975; Crutch and Warrington, 2003; Pulvermuller et al., 2010) and fMRI studies of the healthy brain (Martin, 2007; Martin and Chao, 2001) suggest that semantic processes are at least partially modality- and category-specific. However, the mechanisms by which perceptual and semantic processes interact, and indeed, the extent of any such interaction, have not been fully defined. Furthermore, the brain bases for semantic processing, in health as well as disease, have largely been addressed in the verbal and visual domains. Nonverbal sound processing is an important ‘test case’, both for understanding the organisation of semantic mechanisms that process sensory objects, and more particularly, for understanding semantic disintegration in SD. In the auditory domain, distinct antero-ventral temporal and posterior temporo-parietal networks have been implicated in category-specific semantic processing of animal sounds and tool sounds, respectively (Engel et al., 2009; Lewis et al., 2005; Lewis et al., 2006; Lewis et al., 2011). However, the basis for this apparent dichotomy remains contentious. fMRI evidence in normal subjects (Engel et al., 2009; Leaver and Rauschecker, 2010; Lewis et al., 2011; Staeren et al., 2009) suggests that perceptual and semantic mechanisms interact to represent sound categories. This work has motivated the recent development of cognitive and anatomical models for the processing of ‘auditory objects’ (Griffiths and Warren, 2004; Husain et al., 2004; Goll et al., 2010b). However, the brain mechanisms by which semantic sound categories become established remain controversial. Patients with SD show apperceptive as well as semantic deficits in the visual (Hovius et al., 2003; Joubert et al., 2003) and nonverbal auditory (Goll et al., 2010a) modalities, in keeping with the emerging picture of SD as a brain network disorder rather than a disorder of any single cognitive operation or anatomical area (Fletcher and Warren, 2011). Whereas the study of the normal brain can delineate brain networks engaged in object processing, critical processing mechanisms can only be determined by studying patients with brain damage. SD therefore potentially presents a unique window on the brain networks that represent sound objects and process these representations for meaning: from a neurobiological perspective, SD could be regarded as a model system for assessing essential brain substrates of semantic processing of auditory (as well as other) sensory objects. From a complementary disease perspective, nonverbal sound is an attractive paradigm with which to investigate the brain mechanisms that underpin defective object processing in SD and produce its distinctive clinical phenotype.

Here we investigated brain correlates of auditory object processing in patients with SD compared with healthy older subjects using fMRI. Passive listening to environmental sounds was analysed under perceptual (spectrotemporally filtered versus raw) and semantic (animal versus tool sound category) manipulations, designed to differentiate perceptual and semantic processes and to compare the processing of different sound categories. Our experimental hypotheses were threefold. Firstly, we predicted that auditory perceptual processing would engage similar early auditory and peri-Sylvian regions in SD patients and healthy controls. Secondly, we predicted that SD would be associated with altered activation of anterior temporal lobe regions involved in auditory semantic processing. Finally, we predicted that the SD and healthy control groups would differentially activate separable ventral and dorsal anatomical networks for processing animal sound and tool sound categories respectively.

## Materials and methods

### Subjects

Nine consecutive patients (six males; mean age 64.7 (5.1) years; seven right-handed) meeting consensus criteria (Neary et al., 1998) for a diagnosis of SD were recruited from the tertiary cognitive disorders clinic at the National Hospital for Neurology and Neurosurgery, London, UK. Twenty-two healthy control subjects (12 males; mean age 65.1 (6.8) years; 19 right-handed) with no history of neurological or psychiatric illness also participated. Demographic and general neuropsychological data for all subjects are summarised in Table 1. Patient and control groups did not differ significantly in age (p > 0.9), gender (p > 0.5) or years of education (p > 0.2). In all patients, the syndromic diagnosis of SD was supported by structural brain MRI showing a typical profile of asymmetric (predominantly left-sided) anterior temporal lobe atrophy. All patients had a general neuropsychological assessment confirming a semantic memory deficit relative to the control group; most patients had associated deficits of verbal intelligence, visual object naming and recognition memory, but performed within the normal range on measures of nonverbal intelligence, short term memory and visual object perception, in line with the diagnosis of SD. One patient and two control subjects gave a clinical history of mild peripheral hearing loss. In all subjects, peripheral hearing was assessed at 0.5, 1, 2, 3, 4 kHz using a procedure adapted from a commercial screening audiometry software package (AUDIO-CD™, http://www.digital-recordings.com/audiocd/audio.html). Separate linear regression models were used at each of the frequencies screened to investigate the effect of group on hearing level (with covariates of age and gender), revealing no significant differences between patients and controls (p > 0.05, based upon bootstrapped 95% confidence intervals, bias corrected and accelerated with 2000 replications).

All subjects gave written informed consent to participate and the study was conducted in accord with the guidelines laid down in the Declaration of Helsinki.

### Stimuli

64 animal sounds and 64 tool sounds were selected from on-line databases (e.g. http://www.sonomic.com) according to the following criteria: (i) all sounds were clear representations of a familiar environmental sound source; (ii) tool sounds were associated with a stereotypical action (e.g., using a handsaw to cut wood; using a broom to sweep the floor); (iii) animal sounds were vocalisations with salient harmonic content (animal movement sounds and noisy animal vocalisations, e.g. roaring, were excluded as potentially perceptually confusable with tool sounds). The individual sounds chosen were all unique exemplars, however, particular sound sources were represented in the set more than once (e.g., the sound set contained four distinct exemplars of a cow lowing); the number of exemplars for each type of sound source was in line with the constraints on sound sequence construction (see later) and the relative availability of perceptually distinguishable examples of that target sound in the source database. Individual sounds were shortened to 2 s samples that retained characteristic acoustic features of the sound source. All sound sources used, with their frequencies of occurrence in the set, are listed in Supplementary material on-line, Table S1.

**Fig. 1:**
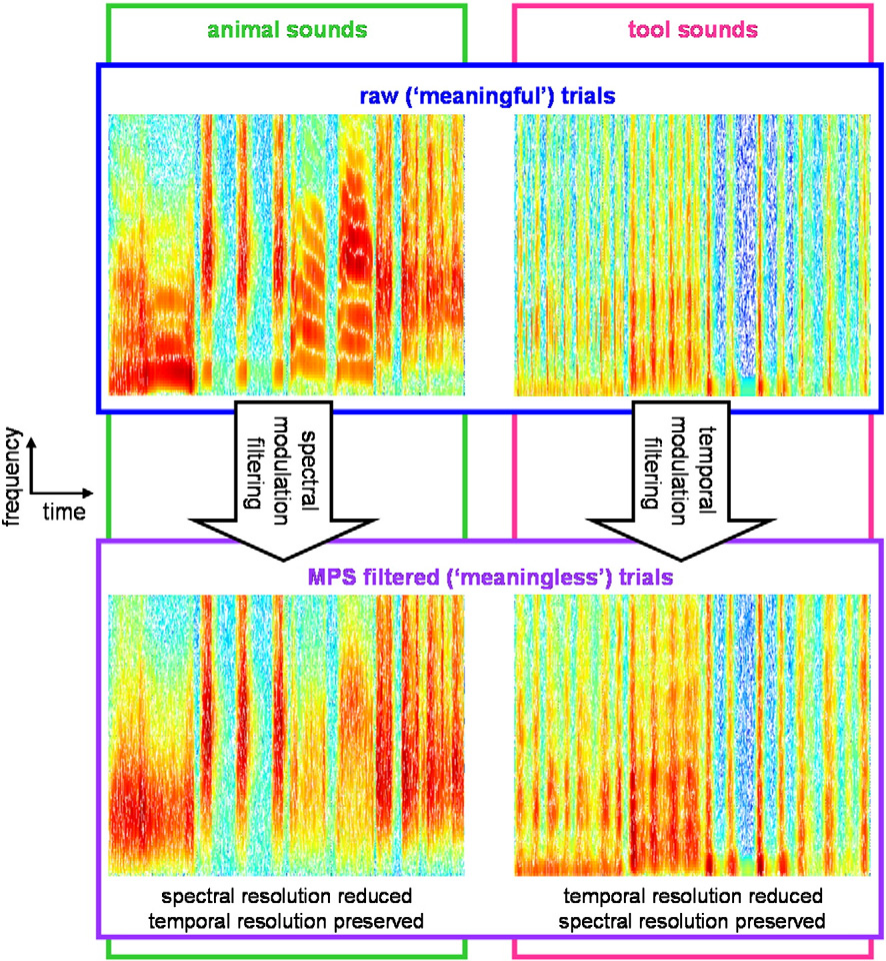
Example spectrograms of tool and animal sounds from ‘meaningful’ and ‘meaningless’ sound conditions. To create ‘meaningless’ trials, ‘meaningful’ (raw) trials were subjected to low-pass modulation power spectrum (MPS) filtering, using a procedure by Elliott and Theunissen (2009); see Section Stimuli for details. Animal trials were filtered in the spectral domain (cut-off point 0.5 cycles/kHz), whilst tool trials were filtered in the temporal domain (cut-off point 4 Hz). Low-pass MPS filtering preserves the overall spectrotemporal content of the sounds, but the resolution of spectral and temporal content is lower in the ‘meaningless’ animal and tool sounds, respectively. This procedure removes cues to sound identity whilst preserving an acoustically complex percept. Sound examples are provided on-line.

To create experimental trials for use during scanning, individual sounds were concatenated into sequences each comprising four different sound sources within the same sound category (tool or animal) with total duration 8 s. A set of 32 ‘meaningful’ trials (16 animal, 16 tool) was created using all 128 raw sounds once. The individual sounds used to form each trial are listed in Supplementary material on-line, Table S2. Meaningful trials were then manipulated to create a matching set of 32 ‘meaningless’ trials, using a procedure developed by (Elliott and Theunissen, 2009) which removes identity information whilst preserving spectrotemporal complexity. This procedure operates over the sound's modulation power spectrum (MPS): the amplitude spectrum of the 2D Fourier transform of the sound's time-frequency representation (spectrogram). Rather than describing acoustic content at any particular point in time (as in a spectrogram), the MPS details modulations over time in both the temporal and spectral domains. MPS filtering enables the removal of energy corresponding to particular temporal and/or spectral modulation ranges (i.e., it reduces spectral or temporal ‘resolution’). For complex broadband sounds, low-pass spectral filtering will preserve the temporal envelope of the original sounds and low-pass temporal modulation filtering will preserve the overall power spectrum of the sound (see Fig. 1). Modulation filtering is a multistep procedure that involves: i) obtaining a time-frequency representation of the sound (here the log of the spectrogram); ii) taking the 2D Fourier Transform (FT) of this representation to obtain the modulation amplitude and phase spectrum; iii) digitally filtering specific temporal-spectral modulations by setting the corresponding amplitudes to zero; iv) inverting the modulation spectrum to obtain a desired time-frequency representation of the modulation filtered sound; and v) inverting the time-frequency representation to obtain the modulation filtered sound. The last step is achieved using a recursive spectrogram inversion algorithm. In order to remove key cues to sound identity for each sound category, animal and tool sounds were low-pass MPS-filtered in the spectral and temporal domains respectively (since vocalisation identity tends to be more dependent on spectral modulation content and tool identity on temporal modulation content); low-pass cut-off values were 0.5 cycles/kHz for animal sounds and 4 Hz for tool sounds. Additionally, to guard against differences between conditions associated with any potential signal-loss effects from the spectrographic inversion in the MPS filtering procedure, the meaningful sounds were subjected to a ‘control’ filtering procedure which consisted of steps (i) and (v) above. Examples of matching meaningful (raw) and meaningless (MPS-filtered) sound trials are available in Supplementary material on-line.

### fMRI paradigm

Four experimental sound conditions each comprising 16 trials were presented in a 2 × 2 factorial design: i) ‘meaningful’ trials comprising sequences of raw animal sounds (mful_a); ii) ‘meaningful’ trials comprising sequences of raw tool sounds (mful_t); iii) ‘meaningless’ trials comprising sequences of MPS-filtered animal sounds (mless_a); iv) ‘meaningless’ trials comprising sequences of MPS-filtered tool sounds (mless_t). An additional low-level baseline condition comprised eight silence trials. Trials were presented in two scanning runs, yielding a total of 72 ∗ 2 = 144 experimental trials. In each run, trials were presented in a random order that was fixed for all subjects. Stimuli were delivered binaurally via electrodynamic headphones (MR Confon GmbH, Magdeburg, http://www.mr-confon.de) at a comfortable sound pressure level (at least 70 dB). In order to minimise cognitive processing demands in the scanner, subjects listened passively to the stimuli with their eyes lightly closed; no in-scanner output task was used.

### Brain image acquisition

All brain images were acquired on a 3 Tesla scanner with 12-channel head coil (Magnetom Trio, Siemens). Single-shot gradient-echo (echoplanar image, EPI) volumes were acquired with the following parameters: 48 oblique transverse slices*;* slice thickness 2 mm; inter-slice gap 1 mm; α = 90^°^; echo time (TE) 30 ms; bandwidth 2298 Hz/pixel; bandwidth in phase-encoding (PE) direction 47.3 Hz/pixel; PE direction anterior-posterior; field of view (FOV) 192 × 192 mm^2^; echo spacing 0.5 ms; matrix size 64 × 64; 13% phase oversampling in the PE direction; fat suppression; descending slice acquisition order. The FOV was positioned to ensure coverage of the entire brain. Blood-oxygen-level-dependent (BOLD) signal losses in the temporal lobes due to susceptibility artifacts were minimised by applying a z-shim gradient moment of + 0.6 mT/m ∗ ms, a slice tilt of − 30^°^, and a positive PE gradient polarity (Weiskopf et al., 2006). To avoid interaction of the stimulus-induced BOLD responses with the response evoked by the gradient noise of the scanner, a ‘sparse-sampling’ acquisition paradigm was used with fixed time-to-repeat of 11.4 s. EPI acquisitions were triggered externally via a laptop running a customised script under MATLAB 7.0 (The Mathworks™). Within each run, 74 brain volumes were acquired for each subject (corresponding to 72 trials, plus two initial dummy scans to allow signal equilibration). To correct for geometric distortions due to B0 field variations, field maps were acquired for each subject after the second run (Cusack et al., 2003; Hutton et al., 2002). For the field map, a double-echo FLASH (GRE) sequence with the following parameters was used: TE1 = 10 ms; TE2 = 12.46 ms; 3 × 3 × 2mm resolution; 1 mm gap.

Volumetric structural MR brain images were acquired using a T1-weighted 3D MDEFT sequence (Deichmann et al., 2004) with the following parameters: sagittal partition direction; 176 partitions; FoV 256 × 240 (or 256 × 256 for subjects with larger heads); matrix 256 × 256; 1^3^ mm resolution; TE 2.48 ms; repetition time 7.92 ms; flip angle 16°; inversion time 910 ms; 50% inversion time ratio; fat saturation angle = 160 degrees; flow suppression angle = 110°; bandwidth = 195 Hz/pix; total acquisition time = 13 min 43 s. Two patients with SD did not have structural MRI acquisitions.

### Out-of-scanner behavioural assessment

Immediately after scanning all subjects completed a novel environmental sound recognition test using 48 of the raw (‘meaningful’) sounds delivered in the scanner (24 animals, 24 tools). Individual sounds (each 2 s in duration) were played in a fixed random order; subjects were asked to match each sound source with its picture from an array of six colour photographs.

### Analysis of fMRI data

Image pre-processing and statistical analyses were performed using Statistical Parametric Mapping software (SPM8©; http://www.fil.ion.ucl.ac.uk/spm). Field maps were reconstructed to obtain voxel displacement maps (VDMs). Images in each scanning run were separately realigned and unwarped using the corresponding VDM to correct for geometric distortions (one SD and one healthy subject did not have a field map; in these subjects, realignment and unwarping were performed without VDM correction, and this methodological difference was accounted for in subsequent statistical modelling). EPI data were then co-registered to the subject's structural MR image, where available.

The resulting native space EPI images were entered into a first-level (within-subject) general linear model (Friston et al., 1994). The evoked hemodynamic response for each stimulus was modelled as a boxcar convolved with a generic haemodynamic response function and sampled at the end of each trial. The design matrix contained both runs, with run-specific regressors for each of the five conditions and six movement-correction parameters obtained from the realign and unwarp steps. Experimental contrasts were constructed as follows: all sound conditions over silence baseline [(mful_a + mful_t + mless_a + mless_t)− 4*silence], to identify brain areas associated with sound processing; meaningless sound conditions over silence baseline [(mless_a + mless_t)–2*silence], to identify areas associated with perceptual processing of spectrotemporally complex sounds; meaningful sound conditions over meaningless sound conditions [(mful_a + mful_t)–(mless_a + mless_t)], to identify areas associated with semantic processing of sounds; the meaningful animal sound condition over the meaningless animal sound condition [m'ful_a–m'less_a], to identify areas associated with semantic processing of animal sounds; the meaningful tool sound condition over the meaningless tool sound condition [m'ful_t–m'less_t], to identify areas associated with semantic processing of tool sounds; the semantic processing of animal sounds over the semantic processing of tool sounds [(mful_a–mless_a)–(mful_t–mless_t)] and the reverse contrast [(mful_t–mless_t)–(mful_a–mless_a)], to identify areas associated with category-specific semantic processing favouring animal and tool sounds, respectively. For each subject, each contrast image was normalised to Montreal Neurological Institute (MNI) space via unified segmentation (Ashburner and Friston, 2005) of the subject's mean functional brain image. Normalised images were smoothed with an isotropic Gaussian kernel of 8 mm full-width at half-maximum.

Individual contrast images were entered into a second-level (between-subjects random effects) model to assess differences between SD and control groups: i.e., the interaction between group and experimental contrast. Inter-subject variation in the use of VDMs during realignment and unwarping was modelled as a nuisance covariate, and variances for SD and control groups were allowed to differ. Voxel clusters were formed at T-contrast height threshold p < 0.001, uncorrected over the whole brain; cluster extents were then assessed at p < 0.05, family-wise error (FWE)-corrected for multiple comparisons over the whole brain. Statistical parametric maps were displayed on a composite structural brain image constructed as the mean of all individual patient and control normalised structural brain images (each individual structural image was normalised to MNI space using subject-specific parameters derived from unified segmentation of the corresponding mean functional brain image).

Comparing groups within experimental contrasts raises a problem of interpretation. For example, regions showing greater activity for patients compared to controls in the contrast [meaningless sounds > silence] could in principle be attributable to increased activity for patients in the ‘forward’ contrast ([meaningless sounds > silence]), or increased activity for controls in the corresponding ‘reverse’ contrast ([silence > meaningless sounds]). This issue is particularly relevant to the functional imaging of patients with neurodegenerative brain disease, who might in principle show either increased or decreased levels of cortical activity relative to healthy controls. Accordingly, a visualisation procedure was employed here to discriminate between these alternative possibilities. Specifically, voxels within the thresholded statistical parametric maps of the contrasts showing higher activity in patients compared to controls (e.g., patients > controls in [meaningless sounds > silence]), were categorised (colour-coded) according to the direction of activation of those voxels in the control group alone; this procedure enabled the disambiguation of voxels that are likely to be driven by greater activation in patients compared to controls in the forward contrast, from those that are likely to be driven by greater activation in controls compared to patients in the reverse contrast.

Data from the category-specific semantic contrast were compared with previously reported patterns of category-specific cortical activity. Local maxima showing preferential bilateral activation for either animal or tool sounds were derived from a previous study by Lewis and colleagues (2005), comprising two ‘animal sound’ foci (in left and right middle superior temporal gyrus, mSTG), and four ‘tool sound’ foci (in left and right posterior lateral sulcus, pLaS, and left and right posterior middle temporal gyrus, pMTG). Coordinates were transformed from Talairach into MNI stereotactic space using a validated conversion algorithm (tal2icbm_spm, http://www.brainmap.org/icbm2tal/; Lancaster et al., 2007). For each subject, effect sizes in the category-specific semantic contrast for the present study were sampled at each of the six foci. The significance of effects within and between groups was assessed using the same model as the main fMRI analysis.

Further separate subanalyses were conducted in the SD group only, incorporating out-of-scanner behavioural data (see Table 1). These subanalyses were designed to determine whether general semantic performance and explicit sound recognition performance were associated with activation in two key contrasts: perceptual processing ([meaningless sounds > silence]) and category-specific semantic processing. Patient scores on a word-picture matching task (the British Picture Vocabulary Scale, Dunn et al., 1982) were used to index general semantic performance, whilst scores on the novel sound-picture matching task were used to index explicit sound recognition performance. In separate subanalyses, data from each key contrast was entered into a second-level linear regression model including one of the two behavioural measures. In each subanalysis, we assessed activation positively correlated with the behavioural measure and activation negatively correlated with the behavioural measure. In addition, effects of varying clinical disease duration across the SD group were assessed in a separate subanalysis. For all subanalyses, results were assessed using cluster-extent statistics at a family-wise error (FWE) corrected threshold of p < 0.05 over the whole brain.

### Voxel-based morphometry

In order to compare activation profiles in SD with the distribution of structural brain damage, regions of reduced grey matter volume in the SD group versus controls were assessed using voxel-based morphometry (VBM). Unified segmentation was applied to all reorientated structural images (22 controls, seven patients) to obtain segmentations of grey matter, white matter and cerebrospinal fluid (CSF). Next, using the subject-specific normalisation parameters derived within the main fMRI analysis, grey matter segments were warped to MNI space with modulation. Normalised images were then smoothed with an isotropic Gaussian kernel of 8 mm full-width at half-maximum. Regional differences in grey matter volume between SD and control groups, incorporating age and total intracranial volume (measured as the sum of grey matter, white matter and CSF segmentations outside of SPM; Whitwell et al., 2001) as nuisance covariates, were assessed using voxel-wise *T*-tests, thresholded leniently at p < 0.001 uncorrected over the whole brain.

In order to determine whether any functional activation differences were solely attributable to regional structural atrophy, we performed a multimodal analysis to assess directly the effects of regional grey matter atrophy on functional activation in the subset of seven SD patients with both functional and structural imaging data (Oakes et al., 2007), assessed (as in the main fMRI analysis) at cluster-extent threshold p < 0.05 FWE-corrected for multiple comparisons over the whole brain. Details of this procedure, sometimes known as biological parametric mapping (Casanova et al., 2007), are presented in Supplementary material on-line.

## Results

### Out-of-scanner behavioural assessment

Most patients performed below the control range on the out-of-scanner sound-picture matching task (see Table 1). The control group performed significantly better for recognition of animal sounds than tool sounds (*t*-test: mean difference = 1.8; 95% confidence interval = 0.8 to 2.9), but the absolute discrepancy in scores between categories was small (Table 1). The SD group was equivalently impaired for recognition of animal and tool sounds (*t*-test: mean difference = 0.3; 95% confidence interval = − 1.9 to 2.6); an analysis of patient scores using the binomial distribution showed that 6/9 patients performed significantly above chance. Taken together these results suggest that any discrepancies in recognition difficulty between sound categories were minor, and that patients were equivalently impaired in the explicit identification of both sound categories.

### fMRI data

**Fig. 2:**
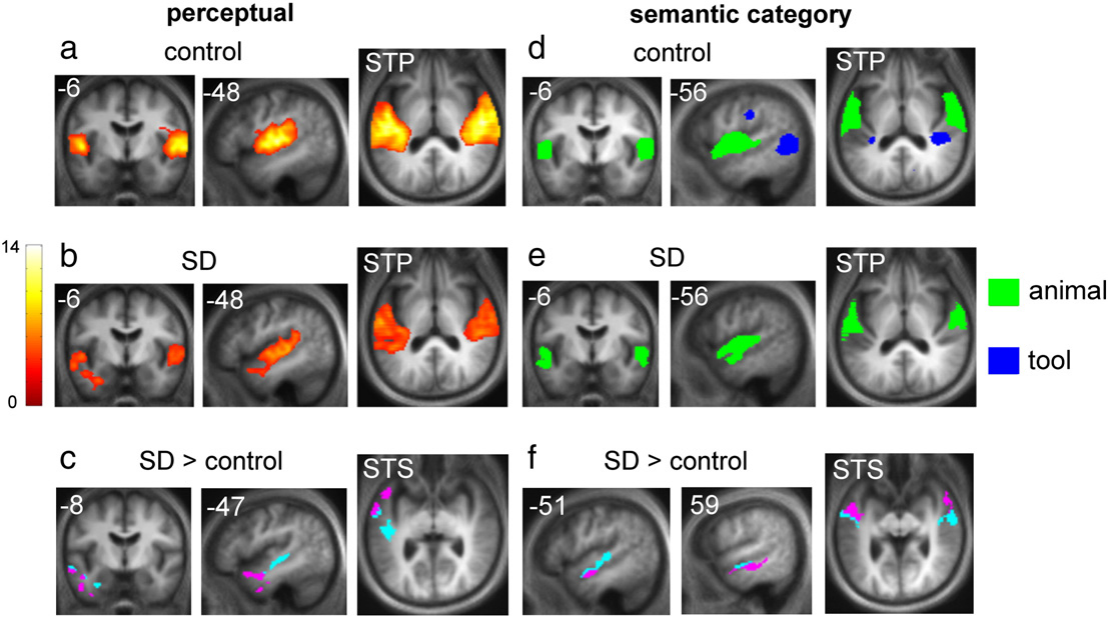
Statistical parametric maps showing activation profiles for perceptual and semantic processing of environmental sounds in healthy controls and patients with semantic dementia. Statistical parametric maps show clusters (formed at whole brain uncorrected height threshold p < 0.001) that are significant at extent threshold p < 0.05, FWE-corrected for multiple comparisons over the whole brain. Maps are rendered on a composite mean normalised structural brain image (see Section Analysis of fMRI data); the left hemisphere is shown on the left for all coronal and axial sections. For sagittal and coronal sections the plane is indicated using MNI coordinates. All axial slices are tilted parallel to the superior temporal plane to show key auditory regions; the anatomical plane of view is indicated. KEY: SD, semantic dementia; STP, superior temporal plane; STS, superior temporal sulcus. The colour key follows. Panels a and b: the colour bar (left) codes voxel-wise T scores for contrast [meaningless sounds > silence]. Panel c: all clusters showing a significant interaction with group (patient > control) for the contrast [all meaningless sounds > silence] are depicted in either magenta or cyan. Magenta codes voxels in which controls alone showed greater activation in the reverse contrast ([silence > meaningful sounds]) than the forwards ([meaningless sounds > silence]) contrast, indicating that the group interaction within these voxels may be driven by greater activation for controls compared to patients in the reverse contrast; however, remaining voxels, coded in cyan, are likely to be driven by greater activation for patients compared to controls in the forwards contrast. Panels d and e: green codes significant clusters in the contrast assessing the category-specific semantic processing favouring animal sounds, [(mful_a–mless_a)–(mful_t–mless_t)]; blue codes significant clusters in the contrast assessing category-specific semantic processing favouring tool sounds, [(mful_t–mless_t)–(mful_a–mless_a)]. Panel f: all clusters showing a significant interaction with group (patient > control) for the contrast assessing category-specific semantic processing favouring animal sounds are depicted in either magenta or cyan. Magenta codes voxels in which controls alone showed greater activation in the reverse contrast (category-specific semantic processing favouring tool sounds) than the forwards (category-specific semantic processing favouring animal sounds) contrast, indicating that the group interaction within these voxels may be driven by greater activation for controls compared to patients in the reverse contrast; however, remaining voxels, coded in cyan, are likely to be driven by greater activation for patients compared to controls in the forwards contrast. See section Analysis of fMRI data for further details.

In describing the fMRI findings we focus on two key contrasts showing areas associated with perceptual processing [meaningless sounds > silence] and areas associated with category-specific semantic processing; and comparisons between the SD and healthy control groups. Significant clusters for the key experimental contrasts (all p < 0.05 after whole-brain FWE correction) are presented in Table 2 and corresponding statistical parametric maps are shown in Fig. 2. Additional contrasts are summarised in Table S3 in Supplementary material on-line.

#### Key perceptual and semantic category contrasts for each group

In the contrast assessing brain areas involved in perceptual processing of sounds ([meaningless sounds > silence]), both the control group and the SD group showed bilateral activation of superior temporal and peri-Sylvian cortices, including medial and lateral Heschl's gyrus (HG), planum temporale (PT), superior temporal gyrus (STG) and sulcus (STS), and posterior insula (Figs. 2a,b; Table 2).

In the contrast assessing category-specific semantic processing favouring animal sounds [(meaningful animal–meaningless animal)–(meaningful tool–meaningless tool)], both the control group and the SD group showed significant bilateral activation in lateral HG and lateral PT and along STG and STS to the temporal poles (Figs. 2d,e; Table 2). In the contrast assessing category-specific semantic processing favouring tool sounds [(meaningful tool–meaningless tool)–(meaningful animal–meaningless animal)], the control group showed significant bilateral activation in a dorsal cortical network including medial PT, posterior insula and MTG (extending to the temporo-occipital junction), precuneus and left inferior parietal cortex; for this contrast no significant activations were identified in the SD group.

**Fig. 3:**
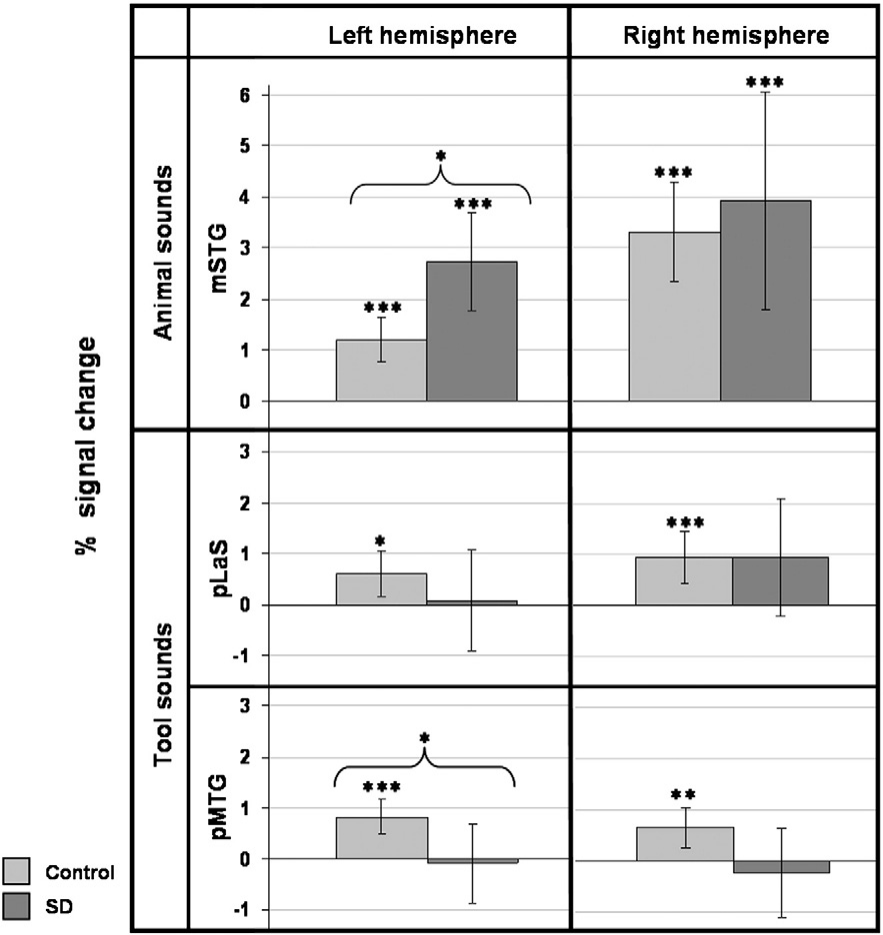
Category-specific contrast effects sampled at previously specified foci of category-specific semantic sound processing. Bars show mean effect sizes (proportionate to percent BOLD signal change) for the control and semantic dementia (SD) patient groups separately for the category-specific semantic contrast at pre-specified foci of category-specific auditory processing (based on Lewis et al., 2005); 95% confidence intervals are also displayed. The upper panels show effects at foci previously associated with animal sound processing in the contrast assessing category-specific semantic processing favouring animal sounds, [(mful_a–mless_a)–(mful_t–mless_t)]; whilst the lower panels show effects at foci previously associated with tool sound processing in the reverse contrast assessing category-specific semantic processing favouring tool sounds, [(mful_t – mless_t) – (mful_a – mless_a)]. Asterisks above bars indicate significance levels for the control and SD groups separately; asterisks above brackets indicate significance levels for between group comparisons. KEY: *p < 0.05; **p < 0.01; ***p < 0.001; mSTG, middle superior temporal gyrus; pLaS, posterior lateral sulcus; pMTG, posterior middle temporal gyrus; SD, semantic dementia.

These data showed a close correspondence with previously reported patterns of category-specific cortical activity during auditory object processing (Lewis et al., 2005; Fig. 3). For the contrast assessing category-specific semantic processing favouring animal sounds, activation in both control and SD groups was significant within the pre-specified animal foci (bilateral middle STG). For the reverse contrast assessing category-specific semantic processing favouring tool sounds, activation in the control group was significant within all pre-specified tool foci (bilateral posterior lateral sulcus, bilateral posterior MTG); however, the SD group did not exhibit significant activity in any of these foci.

#### Key differences between the SD and control groups

For the perceptual contrast, there was a significant interaction with subject group in left STG, STS, middle temporal gyrus (MTG), inferior temporal gyrus (ITG) and temporal pole. A voxel categorisation analysis (Fig. 2c) suggested that group differences in mid and posterior STS and STG were likely to be attributable to a larger effect for patients than controls in the contrast [meaningless sounds > silence], whereas group differences in more inferior and anterior temporal cortex may have been attributable to a larger effect for controls in the reverse contrast [silence > meaningless sounds].

For the key semantic category contrast, there was a significant interaction with group bilaterally in STG, STS and MTG. A voxel categorisation analysis (see Fig. 2f) suggested that group differences in mid STG and STS were likely to be attributable to a larger effect for patients than controls in the contrast assessing category-specific semantic processing favouring animal sounds, whilst group differences more anteriorly in STS and inferiorly in MTG may have been attributable to a larger effect for controls than patients in the reverse contrast assessing category-specific semantic processing favouring tool sounds. There was no evidence for significant activation associated with the reverse interaction (i.e., there was no evidence of a larger effect for patients than controls in the contrast favouring tool sound processing nor a larger effect for controls than patients in the reverse contrast favouring animal sound processing).

#### Other contrasts

Similar activation profiles were observed in additional contrasts assessing all correlates of sound processing ([sound > silence]); semantic processing of sounds combining sound categories ([meaningful sounds > meaningless sounds]); and semantic processing of animal sounds and tool sounds separately ([meaningful animal sounds > meaningless animal sounds]; [meaningful tool sounds > meaningless tool sounds]) (Table S3 on-line). Activation profiles in the contrast assessing all sound processing were similar to the perceptual contrast: both the control group and the SD group showed extensive bilateral activation of superior, anterior and lateral temporal and peri-Sylvian cortices, including medial and lateral HG, PT, STG, STS and posterior insula, with a significant interaction with subject group in bilateral STS, STG, temporal pole, MTG and ITG. In the contrast assessing the semantic processing of sounds combining sound categories, both the control group and the SD group showed extensive bilateral activation throughout superior temporal and peri-Sylvian cortices including lateral HG, PT, STG, and STS; there was a significant interaction with group in midline cerebellum, however no significant group differences were found in any cerebral regions. Contrasts probing the semantic processing of animal sounds and tool sounds separately were similar to the category-specific versions of these contrasts comparing the two semantic categories directly. In the contrast assessing semantic processing of animal sounds alone ([meaningful animal sounds > meaningless animal sounds]), both groups showed extensive bilateral activation extending anteriorly from lateral HG and PT along STG and STS; and for this contrast there was a significant interaction with group in STS, STG and MTG. In the contrast assessing semantic processing of tool sounds alone ([meaningful tool sounds > meaningless tool sounds]), the control group showed significant activation in bilateral posterior superior temporal, insular and right prefrontal cortex; for this contrast, no significant cortical activations were identified in the SD group and there were no significant differences in cortical activation between the groups, though the SD group showed significantly greater activation than controls in caudate nucleus.

#### Effects of behavioural performance and disease duration

In the subanalyses to examine relations between sound processing and out-of-scanner behavioural performance in the SD group, no significant correlations were found between the perceptual contrast [meaningless sounds > silence] and either behavioural measure. There were significant negative correlations with each behavioural measure in the contrast assessing category-specific semantic processing favouring animal sounds, indicating increased activation associated with decreasing behavioural performance (see Table S4 in Supplementary material on-line). These correlations were restricted to posterior areas beyond the activations associated with the category-specific contrast in the main analysis. No negative correlations were found with either behavioural measure in the contrast assessing category-specific semantic processing favouring tool sounds. No significant cerebral activation changes associated with clinical disease duration were identified and in particular the group-wise semantic category interaction remained unaltered when patient-centred disease duration was included as a nuisance covariate.

### VBM data

**Fig. 4:**
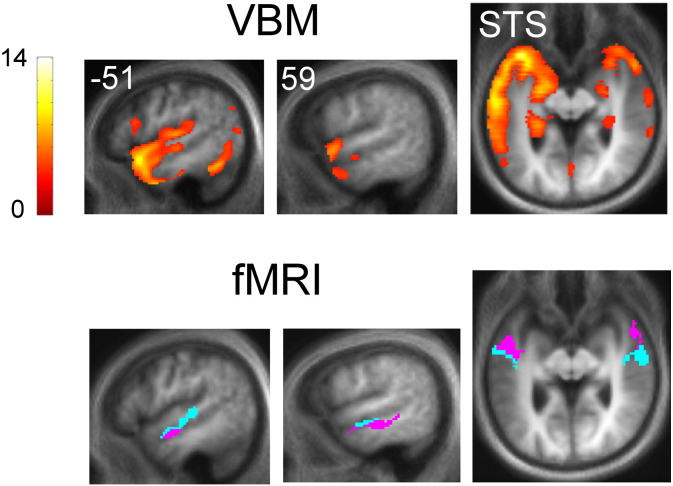
Comparison of structural atrophy and activation maps in semantic dementia. Statistical parametric maps from the voxel-based morphometry (VBM) analysis showing significant (uncorrected p < 0.001 over the whole brain) grey matter loss in the SD group relative to controls are displayed above; below are maps from the fMRI analysis showing a significant interaction with group for the contrast assessing category-specific semantic processing favouring animal sounds (see Fig. 2 legend). VBM and fMRI maps are displayed on matching sections from the same group mean normalised structural image; the plane of the sagittal sections is indicated using MNI coordinates,axial sections have been tilted to run along the superior temporal sulcus (STS) and the left hemisphere is shown on the left. Voxel-wise T score of grey matter change is coded on the colour bar (left). See also Fig. S1 on-line for a direct multimodal comparison between the fMRI and VBM data.

The VBM analysis revealed a typical profile of selective, asymmetric (predominantly left-sided) grey matter atrophy in SD, maximally affecting anterior medial and inferior temporal cortices with less severe involvement of more superior and posterior temporal cortices and some extension to frontal lobe areas. Comparing activation profiles with the distribution of structural atrophy in the SD group (Fig. 4), disease-associated functional changes involved areas of atrophic cortex but extended beyond the zone of maximal structural damage: this was particularly evident for alterations of category-specific semantic processing in the right hemisphere for the SD group compared with the healthy control group. After adjusting the key semantic category-specific contrast directly for local grey matter volume using a voxel-wise covariate (see Supplementary material on-line and Fig. S1), the subgroup of SD patients with both fMRI and structural MRI data showed significantly greater activation than the healthy control group in right mid-STS; no areas of greater activation in controls compared with patients were identified.

## Discussion

Here we have demonstrated altered brain correlates of nonverbal sound analysis in patients with SD compared to healthy individuals. In keeping with our experimental hypotheses, a common bilateral cortical network of superior temporal lobe areas was activated during both perceptual and semantic sound processing in the SD group and in the healthy control group. However, compared with healthy controls, patients with SD showed differential activation of left-sided cortical areas in and adjacent to STS for both perceptual processing of spectrotemporally complex but meaningless sounds and semantic processing of meaningful sound categories (animal sounds versus tool sounds). Additionally, SD patients showed differential activation of left anterior and inferior temporal cortices during perceptual processing, and differential activation of bilateral cortices in MTG during semantic processing. These activation differences in SD were not attributable simply to cortical loss. Whilst imaging modalities must be compared with care, here the activation profile of the key group-wise semantic contrast from the fMRI experiment extended beyond the zone of maximal SD-related atrophy from the VBM analysis (see Fig. 4); and after adjusting for regional grey matter volume directly, there remained increased regional activation in the lateral temporal lobe in the SD group compared with healthy controls (see Fig. S1 on-line). Such increased regional activation compared with healthy controls would be difficult to explain based simply on attenuated processing in atrophic cortex. Moreover, activation changes during category-specific semantic sound processing involved cortical areas distinct from the anatomical correlates of out-of-scanner behavioural measures (indexing general semantic and explicit sound recognition impairment). Taken together, these data suggest that SD leads to an abnormal functional reorganisation of brain mechanisms specifically involved in auditory object analysis.

From the perspective of auditory neurobiology, the finding that cortical substrates for processing particular semantic sound categories are differentially affected by this neurodegenerative disease provides further evidence for the existence of essential category-specific mechanisms of auditory object encoding. In keeping with previous work (Engel et al., 2009; Lewis et al., 2005; Lewis et al., 2006; Lewis et al., 2011; see Fig. 3), the present findings suggest an anatomical and functional dichotomy in the processing of sound categories: animal sounds (vocalisations) were processed in a ventrally directed cortical network whilst tool sounds (movement sounds) were processed in a dorsally directed cortical network. Our findings do not resolve the basis for this dichotomy: it might, however, be based on an association of auditory object representations with different kinds of stored information about sources (animals) versus actions (tools; Engel et al., 2009; Lewis et al., 2005; Lewis et al., 2006; Lewis et al., 2011). Semantic processing of sounds across categories engaged an extensive network centred on the superior temporal lobes in both healthy controls and SD patients, and group differences were identified for semantic processing of particular sound categories considered separately (for animal sounds, in bilateral superior and middle temporal cortices; for tool sounds, in caudate nucleus). However, during category-specific semantic sound processing (i.e., when sound categories were directly compared) group differences were restricted to lateral temporal cortical areas centred on STS. We propose that these areas play a critical role in differentiating sound categories for subsequent analysis in more distributed, functionally connected networks mediating category-specific object identification. This proposal is consistent with evidence in the healthy brain (Caclin and Fonlupt, 2006; Husain et al., 2004; Husain et al., 2006; Leaver and Rauschecker, 2010). In addition, the present findings support previous observations both in patients (Clarke et al., 1996; Goll et al., 2010a) and in healthy individuals (Engel et al., 2009; Leaver and Rauschecker, 2010; Lewis et al., 2011; Staeren et al., 2009), suggesting that perceptual and semantic mechanisms are closely coupled during sound processing. Such a coupling is suggested both by the extensive anatomical overlap of perceptual and semantic processing substrates in the superior temporal lobe in both the healthy and the SD groups here (compare Figs. 2a,b and d,e), and the common involvement of mid and anterior temporal cortices in disease-related alterations affecting both perceptual and semantic levels of analysis (compare Figs. 2c,f). It is unlikely this overlap simply reflects cross-contamination of the semantic category contrast by perceptual stimulus factors, since the contrast here incorporated separate category-specific perceptual baselines closely matched in spectrotemporal complexity to the natural sounds. Rather, we propose that the results delineate a common brain network at the interface of perceptual and semantic mechanisms of sound category representation (Leaver and Rauschecker, 2010). However, the current data do not resolve the relative contributions made by perceptual and semantic mechanisms (which were not constrained by a behavioural task during scanning here), nor the specific effects of the disease process per se on any interaction between these mechanisms.

From a disease perspective, the present findings show that SD gives rise to altered profiles of sensory object processing compared with the healthy brain; and furthermore, the direction of these disease-related alterations is not uniform. Both the perceptual and semantic processing contrasts here were associated with increased activation of mid-temporal cortices in SD patients relative to controls; however, differential activation in more anterior and inferior cortical areas may have been driven by greater activation of these areas by controls in the reverse contrasts (see Fig. 2). This combination of activation changes would fit with structural imaging evidence in SD (Bright et al., 2008; Rohrer et al., 2009): at a given disease stage (and relative to the situation in the healthy brain), reduced cortical function would be associated with more atrophic anterior and inferior temporal regions whilst increased cortical activity would be associated with structurally intact (or less atrophic) posterior and superior regions. It is tempting to conclude that the profile of altered activation in the less affected right hemisphere in the present SD cohort is a marker of regional neuronal dysfunction and a harbinger of tissue destruction; however, a longitudinal analysis would be required to resolve this issue. In principle, disease-related signal increases within the temporal lobes could reflect compensatory over-activation of a damaged object processing network; however, we found no evidence for an association between cortical activity and sound recognition performance in the SD group. Involvement of a putative temporo-polar modality-invariant ‘hub’ has been emphasised as the basis for pan-modal semantic deficits in SD (Lambon Ralph et al., 2010). The present findings suggest a complex derangement of object processing in the anterior temporal lobe with additional involvement of modality-specific cortical regions, consistent with the emerging picture in the healthy brain (Visser et al., 2010; Visser and Lambon Ralph, 2011). Altered object processing associated with SD here was neither cognitively nor anatomically restricted: disease-associated changes were observed at both perceptual and semantic levels of object analysis, and the effect of those functional changes (in particular, failure to activate the dorsal cortical pathway for processing tool sounds) extended beyond the temporal lobes. This interpretation suggests a candidate brain mechanism for neuropsychological defects of object representation in SD (Bozeat et al., 2000; Coccia et al., 2004; Goll et al., 2010a; Luzzi et al., 2007; Pulvermuller et al., 2010; Warrington, 1975). The present data are not necessarily incompatible with a more anterior, multimodal or amodal temporal pole hub; modality-specific object identification must in general be linked with associated knowledge about that object, which could be achieved in the putative hub. Moreover, the current experiment did not employ a correlated behavioural task. Whilst this was intended to avoid potentially confounding task difficulty effects in the patient group, it also follows that the precise level of auditory semantic analysis here was not constrained.

Understanding of the brain basis of neurodegenerative disease has been transformed by the recognition that canonical dementia diseases including SD have syndrome-specific signatures of neural network breakdown that map onto the large-scale network organisation of the healthy brain (Seeley et al., 2009; Zhou et al., 2010; Fletcher and Warren, 2011). The functional changes identified in this fMRI study overlap with anterior and mesial temporal lobe components of the resting brain network previously identified as a signature of SD, but extend beyond that network into uni-modal and multi-modal sensory cortices. Previous functional imaging studies of language processing in SD (Mummery et al., 1999; Wilson et al., 2009) have also demonstrated that disease-related activation changes occur in cortical regions remote from the zone of maximal atrophy, whilst tractographic substrates have been shown using diffusion tensor imaging (Agosta et al., 2010). The present findings show that functional alterations in SD affect widely distributed brain regions that are fundamental for sensory object processing. Additionally, we have shown that auditory object processing engages similar anatomical (especially, superior temporal lobe) regions in patients with SD and in healthy individuals, suggesting that the functional consequences of the disease process as well as the anatomy of disease evolution in SD are partly governed by the neural architecture previously established in the healthy brain. Importantly, however, the functional derangement in SD involves additional cortical regions, leading to a distinctive neural signature not found in healthy individuals. Taken together, these findings suggest that a complete picture of brain network disintegration in neurodegenerative diseases will in general require investigation of the working as well as the resting brain.

There are clear directions for future work arising from this study. The universality of the functional alterations shown here will only be substantiated by parallel studies in other sensory modalities (and across sensory modalities), whilst their disease specificity can only be assessed by parallel studies in other neurodegenerative pathologies. The present investigation did not employ an overt in-scanner task: the behavioural relevance of these activation changes remains to be established. Finally, in an era of intense interest in pathophysiological biomarkers that anticipate tissue destruction (Eidelberg, 2009), there is a need for longitudinal studies to assess how cortical dysfunction in SD and other diseases relates to irreversible cortical loss.

## Figures and Tables

**Table 1:** Subject characteristics and general neuropsychological performance.

	Individual SD patients									SD group	Control group
Case 1	Case 2	Case 3	Case 4	Case 5	Case 6	Case 7	Case 8	Case 9	mean	mean (std. dev.); min.
Sex	m	m	m	m	f	m	f	f	m	6 m, 3 f	10 m, 12 f
Handedness	r	r	r	r	l	r	r	r	l	7 r, 2 l	19 r, 3 l
Age (years)	76	64	63	70	63	63	58	65	60	64.7	65.1 (6.8)
Education (years)	18	10	16	20	10	10	10	12	13	13.2	15.1 (3.8)
Disease duration (years)	3.3	4.3	5.2	4.9	8.4	5.2	6	7	8	5.8	-
MMSE (/30)	29	27	26	24	22	15	12	2	1	17.6	29.3 (0.9); 27
Verbal IQ	78	**55**	**57**	83	**55**	**55**	**55**	**55**	**55**	**61**	-
Performance IQ	119	92	120	133	100	**71**	91	99	114	104	-
RMT — words (z score)	0.7	**− 1.7**	**− 1.7**	− 1.3	**− 1.7**	**− 1.7**	**− 1.7**	**− 1.7**	**− 1.7**	− 1.4	-
RMT — faces (z score)	**− 1.7**	**− 1.7**	− 0.7	**− 1.7**	**− 1.7**	**− 1.7**	**− 1.7**	**− 1.7**	**− 1.7**	− 1.6	-
DS — forwards (z score)	1.5	0.0	− 0.5	**− 1.7**	− 1.0	0.6	**− 1.7**	**− 3.0**	**− 1.7**	− 0.8	-
DS — backwards (z score)	1.0	1.0	1.5	0.3	− 0.3	0.8	**− 3.0**	− 0.8	**− 3.0**	− 0.3	-
Visual object naming (z score)	**− 1.7**	**− 1.7**	**− 1.7**	**− 1.7**	**− 1.7**	**− 1.7**	**− 1.7**	**− 1.7**	**− 1.7**	**− 1.7**	-
Arithmetic (z score)	1.6	0.4	1.6	− 0.6	**− 2.2**	− 1.0	**− 2.3**	**− 2.3**	**− 2.3**	− 0.8	-
Visual object perception (z score)	− 0.3	0.7	0.7	0.3	− 1.3	− 0.7	− 0.7	0.3	− 1.3	− 0.3	-
Word-picture matching (/150)	85	102	88	145	40	77	5	5	5	61	148 (1.1); 146
Snd.–pic. matching — animal (/24)	9	12	14	23	12	10	4*	7*	0*	10	21.7 (1.9); 15
Snd. –pic. matching — tool (/24)	10	17	14	20	8	10	6*	3*	0*	9.8	19.9 (2.1); 16
Synonyms —concrete (z score)	-	**− 5.2**	**− 7.9**	**− 4.1**	**− 6.3**	**− 6.8**	**− 6.8**	-	-	**− 6.2**	-
Synonyms — abstract (z score)	-	**− 3.3**	**− 4.7**	− 0.9	**− 4.7**	**− 4.0**	**− 3.3**	-	-	**− 3.4**	-

Scores were transformed into standardised (IQ or Z) scores based on normative data where available; raw scores are presented for tests where no normative data are available. KEY: bold, patient performance lower than 5th percentile (IQ < 75, Z < − 1.67); underlined, patient performance lower than minimum control score; *, patient performance not significantly different to score expected by chance, calculated using the binomial distribution; -, not tested; Arithmetic, Graded Difficulty Arithmetic test (Jackson and Warrington, 1986); DS, Digit Span test from Wechsler Memory Scale-Revised (WMS-R, Wechsler, 1987); Intelligence, verbal/performance intelligence quotient (Wechsler Abbreviated Scale of Intelligence, Wechsler, 1999); max., maximum; min., minimum; MMSE, Mini-Mental State Examination (Folstein et al., 1975); RMT, Recognition Memory Test (Warrington, 1984); SD, semantic dementia; Snd.–pic. matching, novel sound–picture matching test based upon stimuli from the main fMRI experiment (see Section Out-of-scanner behavioural assessment); std. dev., standard deviation; Synonyms, single word comprehension (Warrington et al., 1998; normative data taken from a local unpublished study by S Connell, EK Warrington, and SJ Crutch); Visual object naming, Graded Naming Test (McKenna and Warrington, 1983); Visual object perception, Object Decision Test from Visual Object and Space Perception Battery (VOSP, Warrington and James, 1991); Word-picture matching, British Picture Vocabulary Scale (Dunn et al., 1982).

**Table 2:** Summary of significant activation clusters in key experimental contrasts.

Contrast	Perceptual						Semantic, favouring animal over tool						Semantic, favouring tool over animal					
[all meaningless sound > silence]						[(mful_a–mless_a)–(mful_t–mless_t)]						[(mful_t–mless_t)–(mful_a–mless_a)]					
	Cluster	Regions	Peaks (x y z)			Hem	Cluster	Regions	Peaks (x y z)			Hem	Cluster	Regions	Peaks (x y z)			Hem
HC	k = 5298 p < 0.001	medHG	− 39	− 24	8	L	k = 1513 p < 0.001	latHG	− 55	− 14	8	L	k = 517 p < 0.001	pMTG	− 57	− 58	0	L
aSTG	− 57	2	− 6	TOJ	− 57	− 70	0
latHG	− 55	− 12	10	k = 265 p = 0.014	insula	− 33	− 32	18
pSTG	− 63	− 26	4	IPL	− 59	− 24	30
k = 218 p = 0.031	precuneus	− 5	− 68	42
k = 5094 p < 0.001	latHG	53	− 16	4	R	k = 1746 p < 0.001	latHG	59	0	4	R	k = 408 p = 0.001	pMTG/STS	63	− 56	12	R
PT	63	− 14	6	TOJ	57	− 66	6
PT	55	− 24	12	k = 342 p = 0.004	insula	35	− 28	18
pSTG	69	− 20	6	PT	41	− 36	18
**SD**	k = 3608 p < 0.001	medHG	− 45	− 24	− 4	L	k = 1713 p < 0.001	aSTS/STG	− 61	− 14	− 4	L	-	-	-	-	-	-
pSTG	− 61	− 22	0	PT	− 53	− 24	2
pSTS/STG	− 63	− 26	4
k = 2311 p < 0.001	PT	51	− 26	10	R	k = 1312 p < 0.001	aSTG	59	0	− 8	R	-	-	-	-	-	-
pSTS/STG	51	− 14	− 4	pSTS/STG	63	− 18	− 10
SD **>** HC	k = 621 p < 0.001	aSTS/MTG	− 55	0	− 24	L	k = 460 p = 0.001	aSTS/MTG	− 53	− 6	− 16	L	-	-	-	-	-	-
k = 199 p = 0.048	ITG	− 51	− 14	− 34	pSTS/STG	− 51	− 26	0
pSTG/STS	− 61	− 20	0	k = 448 p = 0.001	aSTS/MTG	53	2	− 22	R	-	-	-	-	-	-
pSTS/MTG	47	− 24	− 14

Clusters (formed at whole-brain uncorrected height threshold p < 0.001) are significant at extent threshold p < 0.05, FWE-corrected for multiple comparisons over the whole brain. For each cluster, extent (k; voxels), and cluster extent-level significance (FWE p) are shown; to further assist anatomical localisation of the clusters, coordinates of local peaks in MNI stereotactic space (mm) are also shown. KEY: a, anterior; HC, healthy control group; Hem, hemisphere; HG, Heschl's gyrus; ITG, inferior temporal gyrus; lat, lateral; med, medial; mful_a, meaningful animal sound condition, mful_t, meaningful tool sound condition; mless_a, meaningless animal sound condition; mless_t, meaningless tool sound condition; MTG, middle temporal gyrus; p, posterior; PFC, prefrontal cortex; PT, planum temporale; SD, semantic dementia group; STG, superior temporal gyrus; STS, superior temporal sulcus; TOJ, temporo-occipital junction.
