# Supplementary material for: Nonverbal sound processing in semantic dementia: A functional MRI study
Source: Neuroimage. 2012 May 15;61(1):170–80. doi: 10.1016/j.neuroimage.2012.02.045 (PMC3398766; doi:10.1016/j.neuroimage.2012.02.045)
Supplement: Supplementary file 1 — Supplementary materials. [file mmc1.docx]

**Goll JC et al. Nonverbal sound processing in semantic dementia: a functional MRI study - Supplementary material**

**Comparison of functional and structural change in the SD group**

**Methods.** Multimodal analysis (biological parametric mapping) was performed in the subset of 7 SD patients with structural MR brain scans (Oakes et al., 2007, Casanova et al., 2007). The functional data for the group-wise semantic category-specific interaction contrast from these 7 patients and from all healthy control subjects were modelled as the response variable, and the preprocessed VBM data (smoothed modulated normalised GM segmentations) from the same subjects were included as an imaging covariate. Significant group differences in activation were assessed after adjusting for the local GM volume. The analysis was performed using the non-parametric permutation testing software, FSL randomise version 2.5, with 5000 permutations, and variance smoothing with a 3mm standard deviation Gaussian kernel (Nichols & Holmes, 2002). As in the main analysis, clusters were formed at p < 0.001 (or t > 3.4), and multiple comparisons were corrected to control the family-wise error (FWE) for cluster extent at p < 0.05 corrected for the whole brain.

**Results.** After adjusting for structural changes, no regions were found with greater activation in controls than in the SD group at threshold p<0.05 (after cluster-extent FWE correction for multiple comparisons). However, a region in right mid-STS (peak MNI coordinates: 55, -24, -16) was significantly more strongly activated in the SD group than in healthy controls (Figure S1).

**Discussion.** The results in the main manuscript show increased regional activation in SD patients compared to controls, which is unlikely a priori to be attributable purely to structural atrophy. This supplementary multimodal analysis shows that even after adjusting for local GM volume using a voxel-wise covariate, there was significantly increased regional temporal lobe activation in patients that cannot be explained by the local structural information. Including a covariate in the linear model removes all variance that can be linearly explained by it (whether positively or negatively correlated), further strengthening the conclusion that these functional differences are not solely due to local structural atrophy.

**Supplementary references**

Casanova R, ,Srikantha R, Baera A, et al. (2007) Biological parametric mapping: A statistical toolbox for multimodality brain image analysis. NeuroImage 34, 2007, 137-143.

Nichols TE, Holmes AP (2002) Nonparametric permutation tests for functional neuroimaging: A primer with examples. Human Brain Mapping 15: 1–25.

Oakes TR, Fox AS, Johnstone T, Chung MK, Kalin N, Davidson RJ (2007) Integrating VBM into the General Linear Model with voxelwise anatomical covariates. NeuroImage 34, 500-508.

**Figure S1. Effects of regional grey matter adjustment on the comparison of functional activation in the SD and healthy control groups.**


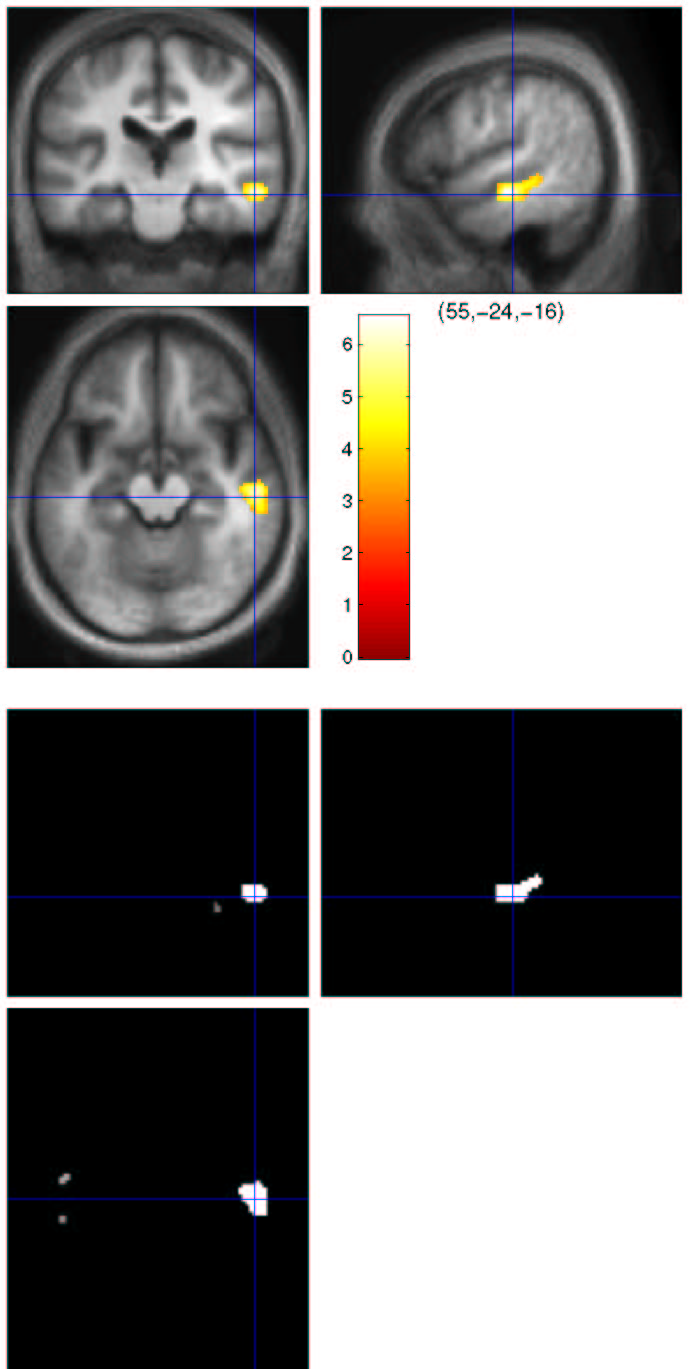


The statistical parametric map (SPM) for the group-wise interaction contrast assessing category-specific semantic processing favouring animal sounds, displayed on the mean normalised structural brain image. Clusters were formed at whole brain uncorrected height threshold p<0.001 and the cluster shown is significant at extent threshold p<0.05, FWE-corrected for multiple comparisons over the whole brain. The voxel-wise activation T score is coded on the colour bar and MNI coordinates of the local peak activation are shown. This SPM is based on the subgroup of seven SD patients with both fMRI and structural MRI data. The adjusted functional data show that these SD patients had significantly greater activation than healthy controls in the right temporal lobe.

**Table S1. Sound sources used to construct experimental trials**

| **Animals** | | **Tools** | |
| --- | --- | --- | --- |
| **Sound source** | **N** | **Sound source** | **N** |
| dog | 8 | slicing food with a knife (slicing) | 6 |
| hen | 6 | using a shovel to move dirt/gravel (shovelling) | 5 |
| chimp | 5 | filing metal (filing) | 4 |
| horse | 5 | hitting with a hammer (hammering) | 4 |
| sheep | 5 | stirring with a spoon/whisk (stirring) | 4 |
| domestic cat | 4 | using a broom/rake to sweep the floor (sweeping) | 4 |
| cow | 4 | writing with pencil on paper/chalk on a blackboard (writing) | 4 |
| duck | 4 | brushing teeth | 3 |
| pig | 4 | sawing wood (sawing) | 3 |
| sealion | 4 | sharpening a knife (sharpening) | 3 |
| big cat | 3 | typing on a computer/typewriter (typing) | 3 |
|  |  | using a stapler (stapling) | 3 |
| bird | 3 | using scissors to cut paper (scissoring) | 3 |
|  |  | chopping wood (chopping) | 2 |
| donkey | 3 | loading a gun (gun loading) | 2 |
|  |  | peeling a vegetable (peeling) | 2 |
| elephant | 3 | sanding wood (sanding) | 2 |
|  |  | using a ratchet (ratchet) | 2 |
| goose | 2 | using a sellotape dispenser (tape) | 2 |
|  |  | locking a door (door locking) | 1 |
| dolphin | 1 | turning a page of a book (page turning) | 1 |
|  |  | using a hole-punch (hole punching) | 1 |

Tool names in parentheses correspond to names used in Table S2. N, number of distinct exemplars of each sound source within the sound set.

**Table S2. Experimental trials**

| **Trial no.** | **Sound no.** | **Animal trials** | **Tool trials** | **Trial no.** | **Sound no.** | **Animal trials** | **Tool trials** |
| --- | --- | --- | --- | --- | --- | --- | --- |
| **1** | **1** | dog | writing | **9** | **1** | pig | sawing |
|  | **2** | donkey | tape |  | **2** | sealion | stirring |
|  | **3** | goose | sweeping |  | **3** | bird | hammering |
|  | **4** | hen | sharpening |  | **4** | dog | slicing |
| **2** | **1** | cow | sanding | **10** | **1** | sheep | sweeping |
|  | **2** | sealion | stirring |  | **2** | goose | sawing |
|  | **3** | chimp | stapling |  | **3** | cow | stirring |
|  | **4** | hen | chopping |  | **4** | dog | sanding |
| **3** | **1** | sheep | filing | **11** | **1** | bird | peeling |
|  | **2** | domestic cat | chopping |  | **2** | cow | scissoring |
|  | **3** | dog | door locking |  | **3** | chimp | filing |
|  | **4** | elephant | sweeping |  | **4** | domestic cat | shovelling |
| **4** | **1** | hen | brushing teeth | **12** | **1** | dog | writing |
|  | **2** | sealion | hammering |  | **2** | sheep | filing |
|  | **3** | dog | scissoring |  | **3** | horse | sharpening |
|  | **4** | chimp | stirring |  | **4** | hen | typing |
| **5** | **1** | duck | writing | **13** | **1** | elephant | gun loading |
|  | **2** | pig | sawing |  | **2** | horse | sharpening |
|  | **3** | chimp | hole punching |  | **3** | dolphin | typing |
|  | **4** | dog | slicing |  | **4** | donkey | shovelling |
| **6** | **1** | big cat | shovelling | **14** | **1** | pig | stapling |
|  | **2** | dog | hammering |  | **2** | horse | slicing |
|  | **3** | chimp | scissoring |  | **3** | elephant | typing |
|  | **4** | sheep | ratchet |  | **4** | hen | brushing teeth |
| **7** | **1** | cow | shovelling | **15** | **1** | duck | brushing teeth |
|  | **2** | duck | slicing |  | **2** | big cat | sharpening |
|  | **3** | domestic cat | ratchet |  | **3** | pig | gun loading |
|  | **4** | bird | sweeping |  | **4** | horse | shovelling |
| **8** | **1** | sheep | filing | **16** | **1** | donkey | writing |
|  | **2** | horse | tape |  | **2** | duck | page turning |
|  | **3** | sealion | hammering |  | **3** | big cat | peeling |
|  | **4** | domestic cat | slicing |  | **4** | hen | stapling |

Experimental trials comprised short sound sequences each derived from four different sound sources (here numbered 1 – 4) all belonging to a particular sound category (animals or tools). All sounds in trials were unique exemplars; however, particular sound sources were represented in the set more than once (e.g., the sound set contained four distinct

exemplars of a cow lowing; see Table S1).

**Table S3. Additional significant activation clusters in experimental contrasts**

| **Contrast** | **all sound > silence** | | | | | | **meaningful > meaningless sound** | | | | | | **meaningful > meaningless animal sound** | | | | | | **meaningful > meaningless tool sound** | | | | | |
| --- | --- | --- | --- | --- | --- | --- | --- | --- | --- | --- | --- | --- | --- | --- | --- | --- | --- | --- | --- | --- | --- | --- | --- | --- |
|  | **Cluster** | **Regions** | **Peaks (x y z)** | | | **Hem** | **Cluster** | **Regions** | **Peaks (x y z)** | | | **Hem** | **Cluster** | **Regions** | **Peaks (x y z)** | | | **Hem** | **k** | **Regions** | **Peaks (x y z)** | | | **Hem** |
| **HC** | k=5326  p<0.001 | latHG | -57 | -14 | 10 | L | k=2572  p<0.001 | medHG | -45 | -22 | 2 | L | k=2535  p<0.001 | PT | -49 | -24 | 4 | L | k=803  p<0.001 | insula | -35 | -32 | 14 | L |
|  |  | medHG | -47 | -22 | 4 |  |  | insula | -37 | -28 | 12 |  |  | PP | -49 | -6 | -4 |  |  | PT | -43 | -34 | 12 |  |
|  | k=946  p<0.001 | PFC | -41 | 14 | 22 |  |  | pSTG/STS | -67 | -32 | 12 |  | k=781  p<0.001 | PFC | -43 | 10 | 24 |  | k=219  p=0.030 | pMTG | -53 | -60 | 2 |  |
|  |  | motor | -51 | -6 | 48 |  | k=918  p<0.001 | PFC | -43 | 18 | 26 |  | k=3195  p<0.001 | PT | 55 | -14 | 2 | R | k=2112  p<0.001 | PT | 45 | -24 | 12 | R |
|  | k=223  p=0.035 | IC | -1 | -40 | -6 |  | k=3688  p<0.001 | PT | 57 | -14 | 4 | R |  | pSTG | 63 | -16 | 0 |  |  | pSTS/STG | 55 | -48 | 6 |  |
|  |  | MGN | -15 | -26 | -4 |  |  | p STS/STG | 63 | -34 | 6 |  |  | aSTG | 57 | 0 | -10 |  | k=459  p=0.001 | PFC | 57 | 28 | 4 |  |
|  | k=5204  p<0.001 | medHG | 53 | -16 | 4 | R | k=835  p<0.001 | PFC | 49 | 32 | 14 |  | k=301  p=0.006 | PFC | 41 | 14 | 24 |  |  |  |  |  |  |  |
|  |  | PT | 61 | -14 | 8 |  |  |  |  |  |  |  |  |  |  |  |  |  |  |  |  |  |  |  |
| **SD** | k=4370  p<0.001 | pSTS/STG | -61 | -22 | 0 | L | k=1941  p<0.001 | aSTG | -59 | -12 | 0 | L | k=2807  p<0.001 | aSTS/STG | -61 | -14 | -2 | L | - | - | - | - | - | - |
|  |  | medHG | -45 | -24 | -4 |  |  | pSTS/STG | -61 | -40 | 4 |  |  | pSTS/STG | -53 | -24 | 0 |  |  |  |  |  |  |  |
|  | k=2789  p<0.001 | pSTS/STG | 53 | -12 | -6 | R | k=2142  p<0.001 | aSTS/STG | 57 | -8 | -10 | R | k=2875  p<0.001 | aSTG | 57 | -2 | -10 | R | - | - | - | - | - | - |
|  |  | PT | 51 | -26 | 10 |  |  | pSTS/STG | 49 | -16 | -2 |  |  | pSTS/STG | 61 | -18 | -10 |  |  |  |  |  |  |  |
|  |  |  |  |  |  |  |  | PT | 51 | -34 | 16 |  |  |  |  |  |  |  |  |  |  |  |  |  |
| **SD > HC** | k=1335  p<0.001 | ITG | -47 | -2 | -40 | L | k=224  p=0.026 | Cerebellum | -9 | -62 | -46 | - | k=453  p=0.001 | pSTS/STG | -47 | -32 | 0 | L | - | - | - | - | - | - |
|  |  | TP | -31 | 12 | -42 |  |  |  | -19 | -58 | -50 |  |  | aSTS/MTG | -55 | 2 | -22 |  |  |  |  |  |  |  |
|  | k=224  p=0.035 | ITG | 55 | -12 | -34 | R |  |  |  |  |  |  | k=393  p=0.001 | pSTS/STG | 59 | -18 | -12 | R | k=204  p=0.040 | Caudate nucleus | 21 | 28 | 6 | R |
|  |  | aSTS/STG | 47 | -2 | -24 |  |  |  | -1 | -64 | -32 |  |  | pMTG | 51 | -28 | -18 |  |  |  |  |  |  |  |
|  |  |  |  |  |  |  |  |  |  |  |  |  |  | aSTS/MTG | 53 | 4 | -26 |  |  |  |  |  |  |  |

Clusters (formed at whole brain uncorrected height threshold p<0.001) are significant at extent threshold p<0.05, FWE-corrected for multiple comparisons over the whole brain. For each cluster, extent (k; voxels), and cluster extent-level significance (FWE p) are shown; to further assist anatomical localisation of the clusters, coordinates of local peaks in MNI stereotactic space (mm) are also shown. KEY: a, anterior; HC, healthy control group; Hem, hemisphere; HG, Heschl’s gyrus; IC, inferior colliculus; ITG, inferior temporal gyrus; lat, lateral; med, medial; MGN, medial geniculate nucleus; MTG, middle temporal gyrus; p, posterior; PFC, prefrontal cortex; PP, planum polare; PT, planum temporale; SD, semantic dementia group; STG, superior temporal gyrus; STS, superior temporal sulcus.

**Table S4. Significant associations with out-of-scanner behavioural measures for category-specific semantic sound processing in the SD group**

| **BPVS** | | | | | |
| --- | --- | --- | --- | --- | --- |
| **Cluster** | **Regions** | **Peaks (x y z)** | | | **Hem** |
| k=84  p=0.010 | occipital pole | 17 | -98 | -2 | R |
| k=76  p=0.017 | cerebellum | -25 | -68 | -20 | L |
|  |  | -29 | -74 | -24 |  |
| **Sound recognition** | | | | | |
| k=104  p=0.002 | TOJ | -29 | -74 | -20 | L |
|  |  | -27 | -68 | -26 |  |
|  |  | -17 | -66 | -18 |  |
| k=83  p=0.009 | posterior cingulate | 7 | -56 | 16 | L/R |
|  |  | -3 | -60 | 14 |  |
| k=80  p=0.011 | posterior ITG | 33 | -62 | -16 | R |
|  |  | 37 | -66 | -22 |  |
| k=60  p=0.046 | visual cortex | 9 | -98 | 0 | R |
|  |  | 15 | -96 | -8 |  |
|  |  | 19 | -96 | 0 |  |

Clusters indicate regions showing inverse associations with out-of-scanner behavioural measures (BPVS score, sound recognition score) in the contrast assessing category-specific semantic processing favouring animal sounds, [(mful_a – mless_a) – (mful_t – mless_t)]. Clusters (formed at whole brain uncorrected height threshold p<0.001) are significant at extent threshold p<0.05, FWE-corrected for multiple comparisons over the whole brain. For each cluster, extent (k; voxels) and cluster extent-level significance (FWE p) are shown; to further assist anatomical localisation of the clusters, coordinates of local peaks in MNI stereotactic space (mm) are also shown. KEY: BPVS, British Picture Vocabulary Scale (Dunn et al., 1982); Hem, hemisphere; ITG, inferior temporal gyrus; SD, semantic dementia; sound recognition, novel sound recognition test (see section 2.5 for details); TOJ, temporo-occipital junction.
